# OnabotulinumtoxinA muscle injection patterns in adult spasticity: a systematic literature review

**DOI:** 10.1186/1471-2377-13-118

**Published:** 2013-09-08

**Authors:** Luba Nalysnyk, Spyridon Papapetropoulos, Philip Rotella, Jason C Simeone, Katharine E Alter, Alberto Esquenazi

**Affiliations:** 1 Epidemiology & Database Analytics, United BioSource Corporation, Lexington, MA, USA; 2 Neurosciences, Medical Affairs, Allergan, Inc., Irvine, CA, USA; 3 Health Economics and Epidemiology, Evidera, Lexington, MA, USA; 4 Rehabilitation Medicine, Mount Washington Pediatric Hospital, Baltimore, MD, USA; 5 Rehabilitation Medicine, NIH, Bethesda, MD, USA; 6 Gait & Motion Analysis Laboratory, MossRehab & Albert Einstein Medical Center, Elkins Park, PA, USA

**Keywords:** Botulinum toxin, OnabotulinumtoxinA, Spasticity, Muscles, Injection patterns

## Abstract

**Background:**

OnabotulinumtoxinA has demonstrated significant benefit in adult focal spasticity. This study reviews the injection patterns (i.e., muscle distribution, dosing) of onabotulinumtoxinA for treatment of adult spasticity, as reported in published studies.

**Methods:**

A systematic review of clinical trials and observational studies published between 1990 and 2011 reporting data on muscles injected with onabotulinumtoxinA in adult patients treated for any cause of spasticity.

**Results:**

28 randomized, 5 nonrandomized, and 37 single-arm studies evaluating 2,163 adult patients were included. The most frequently injected upper-limb muscles were flexor carpi radialis (64.0% of patients), flexor carpi ulnaris (59.1%), flexor digitorum superficialis (57.2%), flexor digitorum profundus (52.5%), and biceps brachii (38.8%). The most frequently injected lower-limb muscles were the gastrocnemius (66.1% of patients), soleus (54.7%), and tibialis posterior (50.5%). The overall dose range reported was 5–200 U for upper-limb muscles and 10–400 U for lower-limb muscles.

**Conclusions:**

The reviewed evidence indicates that the muscles most frequently injected with onabotulinumtoxinA in adults with spasticity were the wrist, elbow, and finger flexors and the ankle plantar flexors. OnabotulinumtoxinA was injected over a broad range of doses per muscle among the studies included in this review, but individual practitioners should be mindful of local regulatory approvals and regulations.

## Background

Spasticity, a phenomenon associated with the upper motor neuron syndrome, was defined by James W. Lance as “a motor disorder characterized by a velocity-dependent increase in tonic stretch reflex (muscle tone) with exaggerated tendon jerks, resulting from hyper-excitability of the stretch reflex as one component of the upper motor neuron syndrome” [1].

The causes of spasticity are heterogeneous. While spasticity following stroke is common, spasticity can also occur in adults following traumatic brain injury, multiple sclerosis, spinal cord injury, and, on some occasions, degenerative central nervous system disorders [2,3]. Spasticity is often classified according to the distribution of body regions affected, which may be focal, regional, or generalized [4,5]. Focal spasticity affects an isolated body part such as the elbow or foot, whereas regional spasticity can affect an entire limb and generalized spasticity affects multiple body areas. Topographical spasticity patterns vary with different etiologies. The distribution of spasticity is important to identify because it has treatment implications [6].

Spasticity-related muscle dysfunction is characterized by muscle hyperactivity/hypertonicity, motor weakness, and, in progressed cases, contracture of adjacent soft tissue [7,8]. Substantial evidence demonstrates that spasticity has a negative impact on patients, causing impairment (e.g., pain, pressure sores, contractures), activity limitation, dependence on caregivers, restriction of participation in social and family life, and decreased overall quality of life [9-11].

Management of spasticity can vary from patient to patient, typically is customized to individual patient needs, and includes a multidisciplinary effort with cooperation and participation of specialists, such as a physiatrist or neurologist, nurses, physical therapists, caregivers, and the patients themselves [12]. Numerous treatments are used to reduce spasticity. Botulinum neurotoxin (BoNT) injections are employed as a focal antispastic agent, usually as part of a broader rehabilitation regimen [4]. In the development of an overall treatment plan, consideration should be given to the treatment goals, including the balance between reduction of spastic hypertonia and preservation of residual motor strength and function [13].

In the United States, there are four botulinum toxin products approved for various indications. Three are serotype A (onabotulinumtoxinA [BOTOX®, Allergan Inc., Irvine, CA, USA]; abobotulinumtoxinA [Dysport®, Ipsen, Paris, France]; and incobotulinumtoxinA [Xeomin®, Merz Pharmaceuticals GmbH, Frankfurt, Germany]), and one is serotype B (rimabotulinumtoxinB [Myobloc®/Neurobloc®, Solstice Neurosciences, San Francisco, CA, USA]). Each differs in molecular structure, formulation, and clinical profile. There is no potency reference standard that is applicable for BoNTs, and each formulation of BoNT is different. Therefore, the units of activity are specific to each product and are not interchangeable with those of any other BoNT [14]. Currently, only onabotulinumtoxinA is approved by the US Food and Drug Administration for the treatment of upper-limb spasticity in adults [15].

Although many studies have been published with regard to the efficacy, safety, and effectiveness of onabotulinumtoxinA for the treatment of spasticity, there is no comprehensive analysis of the literature available that examines the injection patterns of onabotulinumtoxinA. It is therefore the aim of this systematic review to summarize the specific topography of injected muscles, the mean dose of injections per muscle, and the range of doses among patients treated with onabotulinumtoxinA for adult spasticity. It should be noted that “injection patterns” is a broad term that can also include regional topography of injections and techniques for isolating muscles for injection; for the purposes of this review, the term “injection patterns” refers only to the above-mentioned topics of specific topography of injected muscles and dosing.

## Methods

### Literature search

A prospective protocol outlining the methodology of this systematic review was developed and followed. A literature search was performed to identify all English-language clinical trials or observational studies (prospective or retrospective) of onabotulinumtoxinA injections used for the treatment of patients with adult spasticity published since 1990. The methods used to perform this review involved both electronic and manual components, and followed established “best practice” guidelines for systematic review research [16,17]. Searches in MEDLINE and EMBASE were conducted for all studies of BoNT used for treatment of adult spasticity and published between January 1985 and April 2011 to identify any studies of onabotulinumtoxinA. The combination of the following search terms was used: *spastic, spasticity, spasm, hyperreflexia*, *hypertonia, hemiplegia, paralysis.* For details of the specific search terms used, please see Additional file 1. The electronic searches were further supplemented by a manual search of the reference lists of all accepted studies, as well as the reference lists of recent relevant reviews and meta-analyses.

### Study identification

Animal or in vitro studies, studies performed in pediatric populations, and studies reporting treatment with BoNT products other than onabotulinumtoxinA were excluded. The full-text publications of accepted abstracts were reviewed to satisfy all of the following inclusion criteria, regardless of spastic pattern or origin: adult patients with spasticity, treated with onabotulinumtoxinA, and available data on which individual muscles in the upper and/or lower limbs were injected with onabotulinumtoxinA. Included studies were not required to report the number of patients injected or dose parameters for specific muscles, although this data was captured when reported. The agreement of two investigators was required to accept or reject any articles during the review process.

### Data extraction and synthesis

Data elements of interest from each accepted study were extracted to a data extraction form. Extracted information included study-level (e.g., year of publication, geographic region, and study design), patient-level (e.g., sample size, mean age, and gender distribution), and treatment-level (e.g., mean dose and site of onabotulinumtoxinA injection) characteristics. Whenever possible, subgroup data were extracted by spasticity diagnosis from studies enrolling patients with spasticity due to various causes. All injected muscles were considered as reported, regardless of the spastic pattern or origin of spasticity in individuals or patient populations. For studies with patient samples that also included patients treated with other BTX-A formulations, only patients treated with onabotulinumtoxinA were considered. One investigator extracted the data from each study, and a second investigator independently reviewed the extracted data for completeness and accuracy against the original study. Efficacy, safety, and patient-reported outcomes were not assessed in this review and therefore not captured.

Linked studies, which are multiple studies that report outcomes from the same patient sample, in part or in total, were identified during the screening and data extraction phases. When linked studies were identified, only data from the largest, most recent, or most complete report were extracted. However, relevant data from earlier and smaller reports were also used if the report presented pertinent subgroup data not presented in the larger, more complete report. Therefore, all linked studies were considered to be included studies, though each set of linked studies was only counted as one study to avoid double-counting patients.

Descriptive statistics were applied to summarize study-, patient-, and treatment-level data. Information on injected muscles and corresponding onabotulinumtoxinA doses was summarized for individual muscles and muscle groups for upper and lower limbs. Studies reporting on patients treated with onabotulinumtoxinA for both upper- and lower-limb spasticity were included in both respective categories of muscles. Some studies had multiple treatment arms where all patients received onabotulinumtoxinA (with varying doses, patient characteristics, or supplemental treatments such as physiotherapy). All tables include counts of the number of studies (k), the number of onabotulinumtoxinA treatment arms (t), and the number of patients (n) that match a given characteristic, as well as the total number of patients in treatment arms that reported the characteristic (N). To calculate the frequency of injections in individual muscles, the number of all patients/limbs treated with onabotulinumtoxinA in upper limbs or lower limbs, respectively, was used as a denominator, while the numerator for each muscle injected was the number of patients/limbs injected in that particular muscle. Mean doses per muscle were calculated as a mean of all study means, weighted by the number of patients who received injections into each muscle in each study. Note that mean dose may not necessarily account for the full range of doses administered, since dose range was reported in more studies than was mean dose and mean dose could only be tabulated for the subset of studies that explicitly reported it (see semitendinosus, for example). Descriptive summary data, including frequencies of muscles injected, are presented for patients with upper-limb spasticity and lower-limb spasticity, regardless of spasticity origin. Categorical outcomes are presented as proportions, and continuous outcomes are presented using means and ranges.

Information on injected muscles was also analyzed separately by the origin of spasticity (i.e., stroke, traumatic brain injury, spinal cord injury, or multiple sclerosis) using the same methods as described above. These subgroup analyses are presented in Additional files 2, 3, 4 and 5.

Since the focus of this review was not on efficacy and safety, the risk of bias within individual studies was not assessed.

## Results

### Study attrition

**Figure 1:**
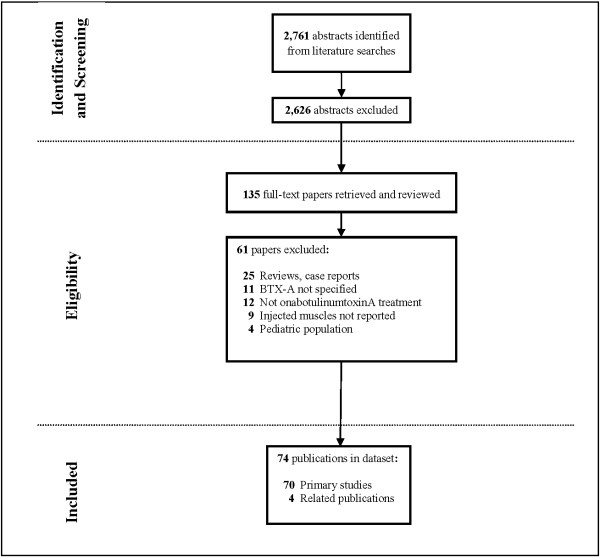
Flow diagram of study attrition.

The entire literature search, including manual bibliography checks, identified 2,761 citations, not including duplicate citations from the various sources. The vast majority of these citations were rejected during abstract screening. Corresponding full papers for 135 abstracts were retrieved for further review and screened against the protocol-specified inclusion criteria. Of the full papers retrieved, 61 were rejected at the full-text review or during data extraction, leaving 74 publications. Among these 74, four pairs of linked publications were identified, from institutions in Turkey [18,19], Italy [20,21], Taiwan [22,23], and France [24,25]. Since only the most informative study was counted from each pair of linked publications, a total of 70 primary studies [2,18,20,23,25-90] pertaining to the treatment of adult spasticity with onabotulinumtoxinA were analyzed. The most common rejection reasons were reviews, not onabotulinumtoxinA treatment, or BoNT formulation not specified. A detailed flow diagram of the study attrition is presented in Figure 1.

### Study characteristics

The majority of the 70 primary studies (comprising 2,163 patients) were conducted in Europe (n = 35) or North America (n = 18), while nine studies were completed in Asia and eight in other geographic locations (Additional file 6). Nearly all included studies (97%) were published since 1995. There were 28 randomized clinical trials, five nonrandomized clinical trials, and 37 single-arm, uncontrolled case series. Sixty-seven studies (96%) were conducted prospectively. Most studies (n = 33) reported only treatment of upper-limb spasticity, 11 studies reported treatment of both upper- and lower-limb spasticity, and 26 studies evaluated patients with lower-limb spasticity. In total, data on upper-limb spasticity was presented in 44 studies comprising 1,670 patients, of which 788 (47.1%) had frequency data reported for onabotulinumtoxinA injections in upper-limb muscles. Data on lower-limb spasticity was reported in 37 studies comprising 1,347 patients, of which 602 (44.7%) had frequency data reported for onabotulinumtoxinA injections in lower-limb muscles. Among the 70 studies (92 treatment arms), the frequency of injections into individual muscles was reported in 55 studies (77 treatment arms), and mean dose per injected muscle was reported in 46 studies (63 treatment arms).

### Baseline patient characteristics

Table 1 presents the summary of baseline characteristics of adult patients treated with onabotulinumtoxinA for upper- or lower-limb spasticity. Among the large majority of studies reporting gender distribution of enrolled patients, males predominated (60.3% and 58.3% for upper- and lower-limb spasticity, respectively). Stroke was the most commonly reported cause of upper- and lower-limb spasticity (66.2% and 62.1%, respectively), while strokes or traumatic brain injuries were combined in several studies as the cause of spasticity in 6.4% (upper limb) and 5.2% (lower limb) of patients. Traumatic brain injury, multiple sclerosis, and spinal cord injuries were reported to be the cause of spasticity in approximately 7% of all patients combined, while in 22.7% of all patients various causes of spasticity were reported. The mean age of patients with spasticity of the upper limb was 51.7 years versus 49.9 years for patients with spasticity of the lower limb. The mean time since disease onset was 46.9 months among patients with spasticity of the upper limb, compared to 53.6 months for patients with spasticity of the lower limb.

**Table 1:** Baseline patient characteristics

**Characteristic**	**Upper-limb spasticity**				**Lower-limb spasticity**			
	**k**	**t**	**n/N**	**Frequency (%)**	**k**	**t**	**n/N**	**Frequency (%)**
**Total**	44	59*	1,670	--	37	47	1,347	--
**Gender**	36	47	1,464	100.0	31	40	1,200	100.0
Male	36	47	883/1,464	60.3	31	40	699/1,200	58.3
Female	36	47	581/1,464	39.7	31	39	501/1,200	41.8
**Spasticity origin**	44	59	1,670	100.0	37	47	1,347	100.0
Stroke only	24	31	1,105/1,670	66.2	16	21	837/1,347	62.1
Stroke/Traumatic brain injury	6	8	107/1,670	6.4	3	3	70/1,347	5.2
Traumatic brain injury only	1	2	21/1,670	1.3	1	1	7/1,347	0.5
Multiple sclerosis	2	3	43/1,670	2.6	5	6	80/1,347	5.9
Spinal cord injury	1	1	28/1,670	1.7	1	2	36/1,347	2.7
Mixed/Other	10	14	366/1,670	21.9	11	14	317/1,347	23.5
	**k**	**t**	**Mean**	**Range of means**	**k**	**t**	**Mean**	**Range of means**
**Age (years)**	39	53	51.7	26 – 66.3	35	45	49.9	26 – 66.3
**Time since onset (months)**	31	43	46.9	4.7 – 120	26	34	53.6	0.35 – 218.4

*k* = Number of studies, *t* = Number of treatment arms, *n* = Number of patients with this characteristic, *N* = Total number of patients in treatment arms reporting characteristic. *The 44 studies that reported on patients with upper-limb spasticity had a total of 59 onabotulinumtoxinA treatment arms; however, only 58 of these arms consisted of at least 1 patient with upper-limb spasticity, as shown in Table 2.

### Characteristics of onabotulinumtoxinA injections

#### Upper-limb muscles

The most frequently injected upper-limb muscles were wrist, finger, and elbow flexors (Table 2). The frequency of onabotulinumtoxinA injections among 788 patients with available data indicates that flexor carpi radialis (64.0% of patients) and flexor carpi ulnaris (59.1%) were the most commonly injected muscles, followed by flexor digitorum superficialis (57.2%), flexor digitorum profundus (52.5%), biceps brachii (38.8%), and flexor pollicis longus (20.4%). Other muscles, including those in the shoulder and forearm, were injected less frequently, ranging from 0.1% to 13.2% of patients.

**Table 2:** Dose range, muscle distribution, and mean dose of onabotulinumtoxinA injections in upper limbs

**Muscles**	**All studies (44 studies, 58 treatment arms)**			**Studies reporting number of patients injected (33 studies, 46 treatment arms)**				**Studies reporting mean dose (26 studies, 39 treatment arms)**			
	**k**	**t**	**Dose range (U)**	**k**	**t**	**n/N**	**Frequency (%)**	**k**	**t**	**n**	**Mean dose (U)**
**Shoulder**											
Infraspinatus	2	2	60	2	2	18/788	2.3	1	1	2	60.0
Shoulder adductors**	1	1	30–100	1	1	8/788	1.0	1	1	8	55.6
Deltoid	1	1	NR	1	1	1/788	0.1	0	0	0	NR
Latissimus dorsi^†^	1	1	50–100	1	1	14/788	1.8	1	1	14	66.1
Levator scapulae	1	1	NR	1	1	1/788	0.1	0	0	0	NR
Paraspinous	1	1	100	1	1	1/788	0.1	1	1	1	100.0
Pectoralis	7	7	30–100	5	5	39/788	4.9	3	3	22	67.3
Rhomboid	1	1	50	1	1	8/788	1.0	1	1	8	50.0
Sternocleidomastoid	1	1	NR	1	1	2/788	0.3	0	0	0	NR
Subscapularis	2	2	100	2	2	26/788	3.3	1	1	10	100.0
Teres major	1	1	40	1	1	1/788	0.1	1	1	1	40.0
Trapezius	2	2	60	2	2	3/788	0.4	1	1	1	60.0
Triceps brachii	5	6	50–100	4	4	10/788	1.3	3	3	9	73.6
**Forearm**											
Extensor carpi radialis	3	4	5–50	3	4	6/788	0.8	2	3	4	17.5
Extensor carpi ulnaris	1	2	5–10	1	2	3/788	0.4	1	2	3	6.7
Extensor digitorum	1	1	NR	1	1	1/788	0.1	0	0	0	NR
Pronator quadrates	2	2	25	1	1	1/788	0.1	0	0	0	NR
Pronator teres	14	16	10–90	10	11	96/788	12.2	6	7	65	33.3
Supinator	1	1	5	1	1	1/788	0.1	1	1	1	5.0
**Elbow flexors**											
Biceps brachii*	30	40	25–200	19	27	306/788	38.8	16	24	263	95.4
Brachialis	10	12	20–100	4	4	27/788	3.4	1	1	1	60.0
Brachioradialis	17	20	15–200	9	11	104/788	13.2	7	9	75	41.1
**Wrist flexors**											
Flexor carpi radialis*	34	45	5–100	24	34	504/788	64.0	18	28	433	49.0
Flexor carpi ulnaris*	34	45	5–100	23	33	466/788	59.1	18	28	420	46.7
Palmaris longus	3	3	20–25	2	2	8/788	1.0	1	1	4	23.8
Wrist flexors^‡^	1	1	50–120	1	1	20/788	2.5	1	1	20	72.8
**Finger flexors**											
Finger flexors^§^	1	1	30–160	1	1	20/788	2.5	1	1	20	97.8
Flexor digitorum profundus*	31	39	5–120	20	27	414/788	52.5	16	23	361	42.0
Flexor digitorum superficialis*	31	40	5–150	20	28	451/788	57.2	16	24	386	50.3
Forearm finger flexor	1	1	80	1	1	5/788	0.6	1	1	5	80.0
Interossei volares	1	1	10–15	1	1	4/788	0.5	1	1	4	11.3
**Thumb**											
Adductor pollicis	7	8	10–20	5	6	102/788	12.9	3	4	93	18.9
Flexor pollicis brevis	1	1	NR	1	1	2/788	0.3	0	0	0	NR
Flexor pollicis longus	20	24	10–35	11	13	161/788	20.4	7	9	132	19.0
Lumbricals	3	4	30	2	2	4/788	0.5	1	1	1	30.0
Opponens	1	1	10	0	0	NR	NR	0	0	0	NR
Opponens pollicis	2	2	NR	1	1	4/788	0.5	0	0	0	NR
**Other muscles**											
Other upper-limb muscles	1	1	NR	0	0	NR	NR	0	0	0	NR
Upper-limb flexors	1	1	50–150	0	0	NR	NR	0	0	0	NR

*Muscles currently approved in the United States for onabotulinumtoxinA injection.

**Deltoid, pectoralis major.

^†^Reported as “dorsalis major”.

^‡^Flexor carpi radialis, palmaris longus, flexor carpi ulnaris.

^§^Flexor pollicis longus, flexor digitorum profundus, flexor digitorum superficialis.

*k* = Number of studies, *t* = Number of treatment arms, *n* = Number of patients injected with onabotulinumtoxinA, *N* = Total number of patients in treatment arms reporting number of patients injected with onabotulinumtoxinA, *NR* = Not reported, *U* = Units.

Overall, the doses of onabotulinumtoxinA injected into upper-limb muscles ranged from 5–200 U among all extracted studies. The mean dose of onabotulinumtoxinA injected into the upper-limb muscles ranged from 5 U in the wrist supinator muscle of the forearm to 100 U injected in the paraspinous and subscapularis muscles of the shoulder. The weighted mean doses varied within muscle groups, although the overall ranges of mean doses were similar among the shoulder muscles (range: 40–100 U), elbow flexors (range: 41.1–95.4 U), wrist flexors (range: 23.8–72.8 U), and finger flexors (range: 11.3–97.8 U). Mean doses injected in the forearm (range: 5.0–33.3 U) and thumb muscles (range: 18.9–30 U) were smaller, as observed by their respective mean dose ranges.

#### Lower-limb muscles

Ankle plantar flexors were the most frequently injected muscles of the lower limb (Table 3). Among 602 patients injected with onabotulinumtoxinA in lower-limb muscles, 66.1% were injected in the gastrocnemius muscles. Over half of the patients were injected in the soleus (54.7%) and the tibialis posterior (50.5%), while 12.5% received injections in the flexor digitorum longus. Less than 10% of patients were injected in other lower-limb muscles, which ranged from 0.2% in the psoas major to 8.3% in the tibialis anterior.

**Table 3:** Dose range, muscle distribution, and mean dose of onabotulinumtoxinA injections in lower limbs

**Muscles**	**All studies (37 studies, 47 treatment arms)**			**Studies reporting number of patients injected (31 studies, 40 treatment arms)**				**Studies reporting mean dose (24 studies, 29 treatment arms)**			
	**k**	**t**	**Dose range (U)**	**k**	**t**	**n/N**	**Frequency (%)**	**k**	**t**	**N**	**Mean dose (U)**
**Hip adductors**											
Adductor longus	4	4	50–400	4	4	26/602	4.3	3	3	17	115.9
Adductor magnus	5	5	20–200	5	5	26/602	4.3	4	4	17	142.4
Adductor brevis	3	3	50–100	3	3	12/602	2.0	3	3	12	91.7
Hip adductors	5	6	50–160	3	3	32/602	5.3	0	0	0	NR
**Hip flexors**											
Iliopsoas	2	2	40–100	2	2	5/602	0.8	0	0	0	NR
Psoas major	1	1	NR	1	1	1/602	0.2	0	0	0	NR
Rectus femoris	6	6	30–200	6	6	42/602	7.0	4	4	32	186.9
**Knee flexors**											
Biceps femoris	6	6	30–200	5	5	34/602	5.6	2	2	6	56.7
Hamstrings	1	1	50–200	0	0	NR	NR	0	0	0	NR
Knee flexors	1	2	NR	0	0	NR	NR	0	0	0	NR
Semimembranosus	8	8	40–200	6	6	41/602	6.8	3	3	4	75.0
Semitendinosus	6	6	30–200	4	4	42/602	7.0	1	1	5	34.0
**Knee extensors**											
Quadriceps	1	1	NR	0	0	NR	NR	0	0	0	NR
Vastus intermedius	1	1	NR	1	1	4/602	0.7	0	0	0	NR
Vastus lateralis	4	4	25–60	3	3	4/602	0.7	2	2	3	38.3
Vastus medialis	3	3	30	2	2	4/602	0.7	1	1	1	30.0
**Ankle plantar flexors**											
Gastrocnemius	31	36	20–320	26	31	398/602	66.1	16	19	246	88.2
Gastrocnemius lateralis	13	14	50–200	12	13	164/602	27.2	9	9	130	86.7
Gastrocnemius medialis	13	14	50–200	12	13	165/602	27.4	9	9	131	86.8
Soleus	25	28	40–240	21	24	329/602	54.7	14	16	233	95.2
Tibialis anterior	7	7	30–150	5	5	50/602	8.3	2	2	37	56.8
Tibialis posterior	24	29	20–200	18	23	304/602	50.5	11	15	218	80.7
**Foot flexors/extensors**											
Extensor hallucis longus	6	6	25–100	5	5	22/602	3.7	4	4	20	71.9
Flexor hallucis longus	7	7	15–95	7	7	33/602	5.5	4	4	22	50.9
Foot flexors	1	2	NR	0	0	NR	NR	0	0	0	NR
Small foot flexors	1	1	50	1	1	5/602	0.8	1	1	5	50.0
**Toe flexors**											
Flexor digitorum longus	10	12	20–125	10	12	75/602	12.5	6	8	50	57.2
Flexor digitorum brevis	6	8	20–100	5	7	25/602	4.2	3	5	18	54.4
Flexor hallucis brevis	2	2	10–15	2	2	3/602	0.5	2	2	3	11.7

*k* = Number of studies, *t* = Number of treatment arms, *n* = Number of patients injected with onabotulinumtoxinA, *N* = Total number of patients in treatment arms reporting number of patients injected with onabotulinumtoxinA, *NR* = Not reported, *U* = Units.

The overall range of doses of onabotulinumtoxinA injected into lower-limb muscles varied greatly, from 10–400 U. Mean doses ranged from 30.0 U in the vastus medialis to 186.9 U in the rectus femoris. Other higher mean doses were also reported among hip adductors (range: 91.7–142.4 U), ankle plantar flexors (range: 56.8–95.2 U), foot flexors and extensors (range: 50.0–71.9 U), and knee flexors (range: 34.0–75.0 U). The lowest mean doses reported among lower-limb muscles were injected in toe flexors (range: 11.7–57.2 U) and knee extensors (range: 30.0–38.3 U).

## Discussion

In this systematic review of the literature, we summarized publications on onabotulinumtoxinA injections in adult patients treated for upper- or lower-limb spasticity. Our goal was to review the injection and dosing patterns reported from various treatment settings including clinical trials and real-world practices, as a practical guidance manuscript of this scope is not currently available in the literature. Seventy-four studies evaluating a total of 2,163 patients were included in this review.

The reviewed evidence indicates that studies vary in terms of geography, study design, sample size, treated patient population, and onabotulinumtoxinA injection practices. The heterogeneity of treatment patterns reported in this review likely reflects real differences in topographical patterns of spasticity. However, it is probable that physician preference, individual interpretation of clinical treatment guidelines, and possibly local health care coverage regulations account for much of the variation reported here. Variation also exists in the injection patterns reported among patients with spasticity from different etiologies (see Additional files 3, 4, 5 and 6).

In the United States, onabotulinumtoxinA is currently indicated for the treatment of upper-limb spasticity in adult patients to reduce the severity of increased muscle tone in elbow flexors (biceps), wrist flexors (flexor carpi radialis and flexor carpi ulnaris), and finger flexors (flexor digitorum profundus and flexor digitorum sublimis). In the reviewed studies, these muscles appear to be commonly injected to treat upper-limb spasticity. In many studies, other upper-limb muscles were also injected, among which the brachialis, brachioradialis, pronator teres, and flexor pollicis longus were most often reported. Among lower-limb muscles, ankle plantar flexors (gastrocnemius, soleus, and tibialis posterior) were most frequently injected. Other lower-limb muscles treated with onabotulinumtoxinA included hip adductors, hip flexors, knee flexors, knee extensors, foot flexors, and toe flexors. Doses of onabotulinumtoxinA varied depending on muscle size, location of injection, severity of spasticity, and whether the injection was the first treatment or was performed taking into account the patients’ responses to previous treatment.

As with any systematic literature review, there are several limitations that should be noted. First, it is important to recognize that not all studies reported detailed information on onabotulinumtoxinA injections. Some only listed muscle groups, others specified individual muscles but did not provide patient counts for injected muscles, and many studies did not report muscle data and thus were excluded from this review. In addition, onabotulinumtoxinA dose information was not reported consistently across all included studies, and thus the estimates of mean dose do not necessarily represent the full range of doses injected in specific muscles. This potentially reduces the accuracy of estimates of the frequency of muscle injection. Second, our findings might be biased by the results from clinical trials, where patients had onabotulinumtoxinA injected into prespecified muscles at specified doses. This may not accurately reflect onabotulinumtoxinA treatment practices in adult patients with spasticity in “real life”. The various aims and scopes of the studies included in this review may have biased the dosage of onabotulinumtoxinA administered in each included study. However, these studies were included because the injection site and/or dosage of onabotulinumtoxinA was discussed in each and the objective of this review was to summarize data on the dose, frequency, and location of intramuscular injections published in the literature, not to review associated efficacy and safety outcomes.

Nevertheless, this literature review indicates that onabotulinumtoxinA is well studied for the treatment of adult spasticity of the upper and lower limbs.

## Conclusions

Although there is a substantial body of published literature on onabotulinumtoxinA in adult spasticity, including systematic reviews and meta-analyses, no prior study, to our knowledge, has systematically summarized injection patterns. This review provides additional information for physicians on varying muscle injection distributions and dosing practices of onabotulinumtoxinA injections in adult spasticity, for both on-label and off-label muscles. These findings indicate that the wrist, elbow, finger flexors, and ankle plantar flexors were most frequently injected with onabotulinumtoxinA in adults with spasticity. The overall dose range of onabotulinumtoxinA injected was 5–200 U for upper-limb muscles and 10–400 U for lower-limb muscles. As regulatory approvals for onabotulinumtoxinA differ across countries, the patterns of injection/treatment presented here are the results of this literature search and are neither an endorsement for use nor a substitution for local regulations.

## Abbreviations

BoNT: Botulinum neurotoxin.

## Competing interests

This research was funded by Allergan, Inc. LN was an employee of United BioSource Corporation, which has received consulting fees from Allergan, Inc., and is currently a full-time employee of Genzyme, a sanofi company. SP was a full-time employee of Allergan, Inc., and is currently a full-time employee of Pfizer Inc. PR is an employee of Evidera, which has received consulting fees from Allergan, Inc. JCS is an employee of Evidera, which has received consulting fees from Allergan, Inc. KEA has received consulting fees and participated on advisory boards for spasticity protocols for Allergan, Inc. AE has received research funding and participated as research advisor for Allergan, Inc., and Ipsen.

## Authors’ contributions

LN was the principal investigator of this study; participated in the study design and coordination; performed the literature review, data collection, and planned statistical analysis; drafted the manuscript; and read and approved the final manuscript. SP was involved in drafting the manuscript and revising it critically for important intellectual content; and read and approved the final manuscript. PR carried out the descriptive analysis of the data, helped to draft the manuscript, and read and approved the final manuscript. JCS participated in the interpretation of the data, helped to draft the manuscript, and read and approved the final manuscript. KEA made substantial contributions to conception, design, and data analysis and interpretation; was involved in critically revising the manuscript; and read and approved the final manuscript. AE made substantial contributions to conception, design, and data analysis and interpretation; was involved in critically revising the manuscript; and read and approved the final manuscript.

## Pre-publication history

The pre-publication history for this paper can be accessed here:

http://www.biomedcentral.com/1471-2377/13/118/prepub

## Supplementary Material

Additional file 1 **Electronic search strategy.** Full, detailed description of the electronic search strategy employed. Click here for file

Additional file 2 **OnabotulinumtoxinA injections for stroke.** Supplemental table presenting subgroup analysis of injected muscles in patients whose spasticity origin was stroke. Click here for file

Additional file 3 **OnabotulinumtoxinA injections for traumatic brain injury.** Supplemental table presenting subgroup analysis of injected muscles in patients whose spasticity origin was traumatic brain injury. Click here for file

Additional file 4 **OnabotulinumtoxinA injections for spinal cord injury.** Supplemental table presenting subgroup analysis of injected muscles in patients whose spasticity origin was spinal cord injury. Click here for file

Additional file 5 **OnabotulinumtoxinA injections for multiple sclerosis.** Supplemental table presenting subgroup analysis of injected muscles in patients whose spasticity origin was multiple sclerosis. Click here for file

Additional file 6 **Study characteristics.** Supplemental table presenting characteristics (publication year, geographic location, study design, study type, and spasticity examined) for all included studies. Click here for file
