# Supplementary material for: OnabotulinumtoxinA muscle injection patterns in adult spasticity: a systematic literature review
Source: BMC Neurol. 2013 Sep 8;13:118. doi: 10.1186/1471-2377-13-118 (PMC3848723; doi:10.1186/1471-2377-13-118)
Supplement: Additional file 1 — Electronic search strategy. Full, detailed description of the electronic search strategy employed. [file 1471-2377-13-118-S1.docx]

# Electronic search strategy

# MEDLINE Search

1. "Muscle spasticity"[MeSH] OR “Spastic” OR "spasticity" OR "Spasm"[MeSH] OR “spasm” OR “spasms” OR "Muscle hypertonia"[MeSH] OR “hypertonia” OR "Hemiplegia"[MeSH] OR “hemiplegia” OR "Paralysis"[MeSH] OR “paralysis” OR "Reflex, Abnormal"[MeSH] OR “hyperreflexia”

2. “onabotulinumtoxinA”[NM] OR “botox” OR “onabotulinumtoxinA” OR “oculinum” OR “vistabel” OR “Botulinum Toxins, Type A”[MeSH] OR “Botulinum Toxins”[MeSH] OR “botulinum” OR “BTX” OR “BoNT”

3. Review[PT] OR Editorial[PT] OR Letter[PT] OR Addresses[PT] OR Bibliography[PT] OR Biography[PT] OR Comment[PT] OR Dictionary[PT] OR Directory[PT] OR Duplicate Publication[PT] OR Festschrift[PT] OR Interview[PT] OR In Vitro[PT] OR Legal Cases[PT] OR Legislation[PT] OR News[PT] OR Newspaper Article[PT] OR Overall[PT] OR Patient Education Handout[PT] OR Periodical Index[PT]

4. #1 AND #2 NOT #3

Limits: Humans, English, Published from 1985/01/01 to 2011/05/01

# EMBASE Search

1. 'spasticity'/exp OR 'spastic' OR 'spasticity' OR 'muscle spasm'/exp OR 'spasm' OR ‘spasms' OR 'muscle hypertonia'/exp OR 'hypertonia' OR 'hemiplegia'/exp OR 'hemiplegia' OR 'paralysis'/exp OR 'paralysis' OR 'hyperreflexia'/exp OR 'hyperreflexia'

2. 'botox' OR 'onabotulinumtoxinA' OR 'oculinum' OR 'vistabel' OR 'botulinum toxin A'/exp OR 'botulinum toxin'/exp OR 'botulinum' OR 'BTX' OR 'BoNT'

3. [conference abstract]/lim OR [conference paper]/lim OR [conference review]/lim OR [editorial]/lim OR [erratum]/lim OR [letter]/lim OR [note]/lim OR [review]/lim OR [short survey]/lim OR 'abstract report':it OR 'book':it OR 'conference paper':it OR 'editorial':it OR 'erratum':it OR 'letter':it OR 'note':it OR 'short survey':it OR 'preliminary communication':it OR 'review':it

4. #1 AND #2 NOT #3

Limits: Humans, English, Published from 1985/01/01 to 2011/05/01
