# Supplementary material for: OnabotulinumtoxinA muscle injection patterns in adult spasticity: a systematic literature review
Source: BMC Neurol. 2013 Sep 8;13:118. doi: 10.1186/1471-2377-13-118 (PMC3848723; doi:10.1186/1471-2377-13-118)
Supplement: Additional file 2 — OnabotulinumtoxinA injections for stroke. Supplemental table presenting subgroup analysis of injected muscles in patients whose spasticity origin was stroke. [file 1471-2377-13-118-S2.docx]

**OnabotulinumtoxinA Injections for Stroke**

| **Injected Muscles** | **All Studies** | | | **Studies Reporting # of Patients Injected** | | | |
| --- | --- | --- | --- | --- | --- | --- | --- |
|  | **k** | **t** | **Dose Range (U)** | **k** | **t** | **n/N** | **Frequency (%)** |
| **Shoulder** |  |  |  |  |  |  |  |
| Shoulder adductors** | 1 | 1 | 30–100 | 1 | 1 | 8/463 | 1.7 |
| Deltoid | 1 | 1 | NR | 1 | 1 | 1/463 | 0.2 |
| Latissimus dorsi^†^ | 1 | 1 | 50–100 | 1 | 1 | 14/463 | 3.0 |
| Infraspinatus | 1 | 1 | NR | 1 | 1 | 16/463 | 3.5 |
| Pectoralis | 4 | 4 | 30–100 | 3 | 3 | 34/463 | 7.3 |
| Rhomboid | 1 | 1 | 50 | 1 | 1 | 8/463 | 1.7 |
| Subscapularis | 2 | 2 | 100 | 2 | 2 | 26/463 | 5.6 |
| Triceps brachii | 1 | 1 | 50 | 1 | 1 | 3/463 | 0.6 |
| **Forearm** |  |  |  |  |  |  |  |
| Extensor carpi radialis | 1 | 1 | 10 | 1 | 1 | 1/463 | 0.2 |
| Extensor carpi ulnaris | 1 | 1 | 10 | 1 | 1 | 1/463 | 0.2 |
| Pronator quadratus | 1 | 1 | 25 | 0 | 0 | NR | NR |
| Pronator teres | 8 | 9 | 10–75 | 6 | 7 | 62/463 | 13.4 |
| **Elbow Flexors** |  |  |  |  |  |  |  |
| Biceps brachii* | 17 | 21 | 25–200 | 12 | 16 | 205/463 | 44.3 |
| Brachialis | 4 | 4 | 50 | 2 | 2 | 14/463 | 3.0 |
| Brachioradialis | 8 | 8 | 20–200 | 5 | 5 | 36/463 | 7.8 |
| **Wrist Flexors** |  |  |  |  |  |  |  |
| Flexor carpi radialis* | 21 | 28 | 5–96.75 | 15 | 22 | 320/463 | 69.1 |
| Flexor carpi ulnaris* | 21 | 28 | 10–93.33 | 14 | 21 | 292/463 | 63.1 |
| Palmaris longus | 3 | 3 | 20–25 | 2 | 2 | 8/463 | 1.7 |
| Wrist flexors^‡^ | 1 | 1 | 50–120 | 1 | 1 | 20/463 | 4.3 |
| **Finger Flexors** |  |  |  |  |  |  |  |
| Finger flexors^§^ | 1 | 1 | 30–160 | 1 | 1 | 20/463 | 4.3 |
| Flexor digitorum profundus* | 21 | 26 | 5–120 | 14 | 19 | 292/463 | 63.1 |
| Flexor digitorum superficialis* | 19 | 24 | 5–77.89 | 12 | 17 | 256/463 | 55.3 |
| Interossei volares | 1 | 1 | 10–15 | 1 | 1 | 4/463 | 0.9 |
| **Thumb** |  |  |  |  |  |  |  |
| Adductor pollicis | 5 | 6 | 10–20 | 3 | 4 | 64/463 | 13.8 |
| Flexor pollicis longus | 13 | 15 | 10–35 | 6 | 8 | 90/463 | 19.4 |
| Lumbricals | 1 | 1 | NR | 1 | 1 | 3/463 | 0.6 |
| Opponens | 1 | 1 | 10 | 0 | 0 | NR | NR |
| Opponens pollicis | 1 | 1 | NR | 0 | 0 | NR | NR |
| **Other Upper** |  |  |  |  |  |  |  |
| Other upper-limb muscles | 1 | 1 | NR | 0 | 0 | NR | NR |
| **Hip Adductors** |  |  |  |  |  |  |  |
| Hip adductors | 1 | 1 | NR | 1 | 1 | 1/324 | 0.3 |
| **Hip Flexors** |  |  |  |  |  |  |  |
| Psoas major | 1 | 1 | NR | 1 | 1 | 1/324 | 0.3 |
| Rectus femoris | 2 | 2 | 30–200 | 2 | 2 | 20/324 | 6.2 |
| **Knee Flexors** |  |  |  |  |  |  |  |
| Biceps femoris | 1 | 1 | NR | 1 | 1 | 1/324 | 0.3 |
| Semimembranosus | 2 | 2 | 60 | 2 | 2 | 3/324 | 0.9 |
| Semitendinosus | 2 | 2 | 40 | 2 | 2 | 3/324 | 0.9 |
| **Knee Extensors** |  |  |  |  |  |  |  |
| Vastus lateralis | 1 | 1 | 60 | 1 | 1 | 1/324 | 0.3 |
| **Ankle Plantarflexors** |  |  |  |  |  |  |  |
| Gastrocnemius | 17 | 21 | 50–320 | 16 | 20 | 257/324 | 79.3 |
| Gastrocnemius lateralis | 10 | 11 | 75–100 | 10 | 11 | 138/324 | 42.6 |
| Gastrocnemius medialis | 9 | 11 | 75–100 | 9 | 11 | 143/324 | 44.1 |
| Soleus | 15 | 17 | 50–125 | 14 | 16 | 227/324 | 70.1 |
| Tibialis anterior | 4 | 4 | 40–150 | 3 | 3 | 40/324 | 12.3 |
| Tibialis posterior | 13 | 17 | 47.8–200 | 12 | 16 | 224/324 | 69.1 |
| **Foot Flexors** |  |  |  |  |  |  |  |
| Extensor hallucis longus | 1 | 1 | 25–100 | 1 | 1 | 11/324 | 3.4 |
| Flexor hallucis longus | 4 | 4 | 25–75 | 4 | 4 | 19/324 | 5.9 |
| **Foot Muscles** |  |  |  |  |  |  |  |
| Flexor digitorum longus | 6 | 8 | 50–100 | 6 | 8 | 37/324 | 11.4 |
| Flexor digitorum brevis | 2 | 4 | 50–100 | 2 | 4 | 11/324 | 3.4 |

k = Number of studies; t = Number of treatment arms; n = Number of patients injected with onabotulinumtoxinA; N = Total number of patients in treatment arms reporting number of patients injected with onabotulinumtoxinA; NR = Not reported; U = Units.
