# Supplementary material for: OnabotulinumtoxinA muscle injection patterns in adult spasticity: a systematic literature review
Source: BMC Neurol. 2013 Sep 8;13:118. doi: 10.1186/1471-2377-13-118 (PMC3848723; doi:10.1186/1471-2377-13-118)
Supplement: Additional file 3 — OnabotulinumtoxinA injections for traumatic brain injury. Supplemental table presenting subgroup analysis of injected muscles in patients whose spasticity origin was traumatic brain injury. [file 1471-2377-13-118-S3.docx]

**OnabotulinumtoxinA Injections for Traumatic Brain Injury**

| **Injected Muscles** | **All Studies** | | | **Studies Reporting # of Patients Injected** | | | |  |
| --- | --- | --- | --- | --- | --- | --- | --- | --- |
|  | **k** | **t** | **Dose Range (U)** | **k** | **t** | **n/N** | **Frequency (%)** | |
| **Shoulder** |  |  |  |  |  |  |  | |
| Deltoid | 1 | 1 | NR | 1 | 1 | 1/9 | 11.1 | |
| Sternocleidomastoid | 1 | 1 | NR | 1 | 1 | 1/9 | 11.1 | |
| Trapezius | 1 | 1 | NR | 1 | 1 | 1/9 | 11.1 | |
| **Forearm** |  |  |  |  |  |  |  | |
| Extensor carpi radialis | 1 | 1 | NR | 1 | 1 | 2/9 | 22.2 | |
| Extensor digitorum | 1 | 1 | NR | 1 | 1 | 1/9 | 11.1 | |
| Pronator quadratus | 1 | 1 | NR | 1 | 1 | 1/9 | 11.1 | |
| Pronator teres | 1 | 1 | NR | 1 | 1 | 2/9 | 22.2 | |
| **Elbow Flexors** |  |  |  |  |  |  |  | |
| Biceps brachii | 2 | 3 | NR | 1 | 1 | 3/9 | 33.3 | |
| Brachialis | 2 | 3 | NR | 1 | 1 | 3/9 | 33.3 | |
| Brachioradialis | 1 | 1 | NR | 1 | 1 | 6/9 | 66.7 | |
| **Wrist Flexors** |  |  |  |  |  |  |  | |
| Flexor carpi radialis | 2 | 3 | NR | 1 | 1 | 2/9 | 22.2 | |
| Flexor carpi ulnaris | 2 | 3 | NR | 1 | 1 | 2/9 | 22.2 | |
| **Finger Flexors** |  |  |  |  |  |  |  | |
| Flexor digitorum profundus | 2 | 3 | NR | 1 | 1 | 5/9 | 55.6 | |
| Flexor digitorum superficialis | 2 | 3 | NR | 1 | 1 | 5/9 | 55.6 | |
| **Thumb Flexors** |  |  |  |  |  |  |  | |
| Adductor pollicis | 1 | 1 | NR | 1 | 1 | 2/9 | 22.2 | |
| Flexor pollicis brevis | 1 | 1 | NR | 1 | 1 | 1/9 | 11.1 | |
| Flexor pollicis longus | 1 | 2 | NR | 0 | 0 | NR | NR | |
| Opponens pollicis | 1 | 1 | NR | 1 | 1 | 1/9 | 11.1 | |
| **Knee Flexors** |  |  |  |  |  |  |  | |
| Biceps femoris | 1 | 1 | 85 | 1 | 1 | 1/19 | 5.3 | |
| Semimembranosus | 1 | 1 | NR | 1 | 1 | 2/19 | 10.5 | |
| Semitendinosus | 2 | 2 | 30 | 2 | 2 | 3/19 | 15.8 | |
| **Ankle Plantarflexors** |  |  |  |  |  |  |  | |
| Gastrocnemius | 3 | 3 | 100 | 3 | 3 | 15/19 | 78.9 | |
| Gastrocnemius lateralis | 1 | 1 | 100 | 1 | 1 | 7/19 | 36.8 | |
| Gastrocnemius medialis | 2 | 2 | 100 | 2 | 2 | 9/19 | 47.4 | |
| Soleus | 2 | 2 | 100 | 2 | 2 | 13/19 | 68.4 | |
| Tibialis anterior | 1 | 1 | NR | 1 | 1 | 1/19 | 5.3 | |
| Tibialis posterior | 2 | 2 | NR | 1 | 1 | 3/19 | 15.8 | |
| **Foot Flexors** |  |  |  |  |  |  |  | |
| Extensor hallucis longus | 1 | 1 | NR | 1 | 1 | 1/19 | 5.3 | |
| Flexor hallucis longus | 1 | 1 | NR | 1 | 1 | 1/19 | 5.3 | |
| **Toe Flexors** |  |  |  |  |  |  |  | |
| Flexor digitorum longus | 1 | 1 | NR | 1 | 1 | 1/19 | 5.3 | |

k = Number of studies; t = Number of treatment arms; n = Number of patients injected with onabotulinumtoxinA; N = Total number of patients in treatment arms reporting number of patients injected with onabotulinumtoxinA; NR = Not reported; U = Units.
