# Supplementary material for: OnabotulinumtoxinA muscle injection patterns in adult spasticity: a systematic literature review
Source: BMC Neurol. 2013 Sep 8;13:118. doi: 10.1186/1471-2377-13-118 (PMC3848723; doi:10.1186/1471-2377-13-118)
Supplement: Additional file 4 — OnabotulinumtoxinA injections for spinal cord injury. Supplemental table presenting subgroup analysis of injected muscles in patients whose spasticity origin was spinal cord injury. [file 1471-2377-13-118-S4.docx]

**OnabotulinumtoxinA Injections for Spinal Cord Injury**

| **Injected Muscles** | **All Studies** | | | **Studies Reporting # of Patients Injected** | | | |
| --- | --- | --- | --- | --- | --- | --- | --- |
|  | **k** | **t** | **Dose Range (U)** | **k** | **t** | **n/N** | **Frequency (%)** |
| **Shoulder** |  |  |  |  |  |  |  |
| Infraspinatus | 1 | 1 | 60 | 1 | 1 | 2/29 | 6.9 |
| Paraspinous | 1 | 1 | 100 | 1 | 1 | 1/29 | 3.4 |
| Pectoralis | 1 | 1 | 60–80 | 1 | 1 | 4/29 | 13.8 |
| Teres major | 1 | 1 | 40 | 1 | 1 | 1/29 | 3.4 |
| Trapezius | 1 | 1 | 60 | 1 | 1 | 1/29 | 3.4 |
| Triceps brachii | 1 | 1 | 60–100 | 1 | 1 | 4/29 | 13.8 |
| **Forearm** |  |  |  |  |  |  |  |
| Extensor carpi radialis | 1 | 1 | 50 | 1 | 1 | 1/29 | 3.4 |
| Pronator teres | 1 | 1 | 10–90 | 1 | 1 | 3/29 | 10.3 |
| **Elbow Flexors** |  |  |  |  |  |  |  |
| Biceps brachii | 1 | 1 | 30–90 | 1 | 1 | 3/29 | 10.3 |
| Brachialis | 1 | 1 | 60 | 1 | 1 | 1/29 | 3.4 |
| Brachioradialis | 1 | 1 | 20–40 | 1 | 1 | 3/29 | 10.3 |
| **Wrist Flexors** |  |  |  |  |  |  |  |
| Flexor carpi radialis | 1 | 1 | 20–60 | 1 | 1 | 9/29 | 31.0 |
| Flexor carpi ulnaris | 1 | 1 | 20–50 | 1 | 1 | 8/29 | 27.6 |
| **Finger Flexors** |  |  |  |  |  |  |  |
| Flexor digitorum profundus | 1 | 1 | 30–70 | 1 | 1 | 4/29 | 13.8 |
| Flexor digitorum superficialis | 1 | 1 | 30–60 | 1 | 1 | 10/29 | 34.5 |
| **Thumb** |  |  |  |  |  |  |  |
| Flexor pollicis longus | 1 | 1 | 20–30 | 1 | 1 | 3/29 | 10.3 |
| Lumbricals | 1 | 1 | 30 | 1 | 1 | 1/29 | 3.4 |
| Lumbricals | 1 | 1 | 30 | 1 | 1 | 1/29 | 3.4 |
| **Hip Adductors** |  |  |  |  |  |  |  |
| Adductor longus | 2 | 2 | 50–400 | 2 | 2 | 6/29 | 20.7 |
| Adductor magnus | 2 | 2 | 50–100 | 2 | 2 | 4/29 | 13.8 |
| Adductor brevis | 2 | 2 | 50 | 2 | 2 | 2/29 | 6.9 |
| **Knee Flexors** |  |  |  |  |  |  |  |
| Semimembranosus | 2 | 2 | 100 | 2 | 2 | 2/29 | 6.9 |
| **Knee Extensors** |  |  |  |  |  |  |  |
| Vastus lateralis | 1 | 1 | 30 | 1 | 1 | 1/29 | 3.4 |
| Vastus medialis | 1 | 1 | 30 | 1 | 1 | 1/29 | 3.4 |
| **Ankle Plantarflexors** |  |  |  |  |  |  |  |
| Gastrocnemius | 1 | 1 | 75–200 | 1 | 1 | 7/29 | 24.1 |
| Gastrocnemius lateralis | 1 | 1 | 75–200 | 1 | 1 | 7/29 | 24.1 |
| Gastrocnemius medialis | 1 | 1 | 75–200 | 1 | 1 | 7/29 | 24.1 |
| Soleus | 1 | 1 | 40–100 | 1 | 1 | 5/29 | 17.2 |
| Tibialis anterior | 1 | 1 | 100 | 1 | 1 | 2/29 | 6.9 |
| Tibialis posterior | 1 | 1 | 50–60 | 1 | 1 | 2/29 | 6.9 |
| **Foot Flexors** |  |  |  |  |  |  |  |
| Extensor hallucis longus | 1 | 1 | 50 | 1 | 1 | 2/29 | 6.9 |
| Flexor hallucis longus | 1 | 1 | 80 | 1 | 1 | 1/29 | 3.4 |
| **Toe Flexors** |  |  |  |  |  |  |  |
| Flexor hallucis brevis | 1 | 1 | 10 | 1 | 1 | 1/29 | 3.4 |

k = Number of studies; t = Number of treatment arms; n = Number of patients injected with onabotulinumtoxinA; N = Total number of patients in treatment arms reporting number of patients injected with onabotulinumtoxinA; U = Units.
