# Supplementary material for: OnabotulinumtoxinA muscle injection patterns in adult spasticity: a systematic literature review
Source: BMC Neurol. 2013 Sep 8;13:118. doi: 10.1186/1471-2377-13-118 (PMC3848723; doi:10.1186/1471-2377-13-118)
Supplement: Additional file 5 — OnabotulinumtoxinA injections for multiple sclerosis. Supplemental table presenting subgroup analysis of injected muscles in patients whose spasticity origin was multiple sclerosis. [file 1471-2377-13-118-S5.docx]

**OnabotulinumtoxinA Injections for Multiple Sclerosis**

| **Injected Muscles** | **All Studies** | | | **Studies Reporting # of Patients Injected** | | | |
| --- | --- | --- | --- | --- | --- | --- | --- |
|  | **k** | **t** | **Dose Range (U)** | **k** | **t** | **n/N** | **Frequency (%)** |
| **Elbow Flexors** |  |  |  |  |  |  |  |
| Biceps brachii | 1 | 1 | 100 | 1 | 1 | 1/44 | 2.3 |
| Brachioradialis | 1 | 1 | 100 | 1 | 1 | 1/44 | 2.3 |
| **Wrist Flexors** |  |  |  |  |  |  |  |
| Flexor carpi radialis | 1 | 2 | 100 | 1 | 2 | 38/44 | 86.4 |
| Flexor carpi ulnaris | 2 | 3 | 80–100 | 2 | 3 | 43/44 | 97.7 |
| **Finger Flexors** |  |  |  |  |  |  |  |
| Flexor digitorum superficialis | 1 | 2 | 100 | 1 | 2 | 38/44 | 86.4 |
| Forearm finger flexor | 1 | 1 | 80 | 1 | 1 | 5/44 | 11.4 |
| **Hip Adductors** |  |  |  |  |  |  |  |
| Adductor longus | 2 | 2 | 70–100 | 2 | 2 | 12/57 | 21.1 |
| Adductor magnus | 2 | 2 | 100–200 | 2 | 2 | 11/57 | 19.3 |
| Adductor brevis | 1 | 1 | 100 | 1 | 1 | 10/57 | 17.5 |
| Hip adductors | 1 | 1 | NR | 0 | 0 | NR | NR |
| Rectus femoris | 1 | 1 | 50 | 1 | 1 | 1/57 | 1.8 |
| **Knee Flexors** |  |  |  |  |  |  |  |
| Biceps femoris | 1 | 1 | 55–90 | 1 | 1 | 3/57 | 5.3 |
| Semimembranosus | 2 | 2 | 40 | 1 | 1 | 1/57 | 1.8 |
| Semitendinosus | 2 | 2 | 30 | 1 | 1 | 3/57 | 5.3 |
| Knee flexors | 1 | 1 | NR | 0 | 0 | NR | NR |
| **Knee Extensors** |  |  |  |  |  |  |  |
| Vastus lateralis | 2 | 2 | 25 | 1 | 1 | 1/57 | 1.8 |
| Vastus medialis | 1 | 1 | NR | 0 | 0 | NR | NR |
| **Ankle Plantarflexors** |  |  |  |  |  |  |  |
| Gastrocnemius | 4 | 5 | 25–120 | 3 | 4 | 44/57 | 77.2 |
| Soleus | 3 | 4 | 120 | 2 | 3 | 43/57 | 75.4 |
| Tibialis posterior | 2 | 3 | NR | 1 | 2 | 38/57 | 66.7 |
| **Foot Flexors** |  |  |  |  |  |  |  |
| Foot flexors | 1 | 1 | NR | 0 | 0 | NR | NR |
| Small foot flexors | 1 | 1 | 50 | 1 | 1 | 5/57 | 8.8 |

k = Number of studies; t = Number of treatment arms; n = Number of patients injected with onabotulinumtoxinA; N = Total number of patients in treatment arms reporting number of patients injected with onabotulinumtoxinA; NR = Not reported; U = Units.
