# Supplementary material for: OnabotulinumtoxinA muscle injection patterns in adult spasticity: a systematic literature review
Source: BMC Neurol. 2013 Sep 8;13:118. doi: 10.1186/1471-2377-13-118 (PMC3848723; doi:10.1186/1471-2377-13-118)
Supplement: Additional file 6 — Study characteristics. Supplemental table presenting characteristics (publication year, geographic location, study design, study type, and spasticity examined) for all included studies. [file 1471-2377-13-118-S6.docx]

**Study Characteristics**

|  | **All Studies** | | | **Studies of Upper-Limb Spasticity** | | | **Studies of Lower-Limb Spasticity** | | |
| --- | --- | --- | --- | --- | --- | --- | --- | --- | --- |
|  | **k** | **t** | **N** | **k** | **t** | **N** | **k** | **t** | **N** |
| **Totals** | 70 | 92 | 2163 | 44 | 59 | 1670 | 37 | 47 | 1347 |
| **Publication Year** |  |  |  |  |  |  |  |  |  |
| 1985–1994 | 2 | 2 | 22 | 0 | 0 | 0 | 2 | 2 | 22 |
| 1995–2005 | 35 | 48 | 871 | 23 | 32 | 619 | 17 | 23 | 367 |
| 2006–2011 | 33 | 42 | 1270 | 21 | 27 | 1051 | 18 | 22 | 958 |
| **Geographic Location** |  |  |  |  |  |  |  |  |  |
| North America | 18 | 30 | 951 | 15 | 26 | 917 | 6 | 8 | 616 |
| Europe | 35 | 41 | 795 | 19 | 21 | 472 | 21 | 26 | 525 |
| Asia | 9 | 10 | 280 | 7 | 8 | 202 | 3 | 3 | 85 |
| Other | 8 | 11 | 137 | 3 | 4 | 79 | 7 | 10 | 121 |
| **Study Design** |  |  |  |  |  |  |  |  |  |
| RCT | 28 | 46 | 1263 | 16 | 28 | 1013 | 16 | 24 | 869 |
| nRCT | 5 | 9 | 161 | 4 | 7 | 141 | 3 | 5 | 75 |
| UCS | 37 | 37 | 739 | 24 | 24 | 516 | 18 | 18 | 403 |
| **Study Type** |  |  |  |  |  |  |  |  |  |
| Prospective | 67 | 89 | 2025 | 42 | 57 | 1542 | 34 | 44 | 1209 |
| Retrospective | 3 | 3 | 138 | 2 | 2 | 128 | 3 | 3 | 138 |
| **Spasticity Examined** |  |  |  |  |  |  |  |  |  |
| Upper limbs only | 33 | 45 | 816 | 33 | 45 | 816 | 0 | 0 | 0 |
| Lower limbs only | 26 | 33 | 493 | 0 | 0 | 0 | 26 | 33 | 493 |
| Upper and lower limbs | 11 | 14 | 854 | 11 | 14 | 854 | 11 | 14 | 854 |

k = Number of studies; t = Number of treatment arms; N = Total number of patients in treatment arms reporting number of patients injected with onabotulinumtoxinA.
